# Pediatric non–Down’s syndrome acute megakaryoblastic leukemia patients in China: A single center's real-world analysis

**DOI:** 10.3389/fonc.2022.940725

**Published:** 2022-10-04

**Authors:** Aoli Zhang, Lipeng Liu, Suyu Zong, Xiaoyan Chen, Chao Liu, Lixian Chang, Xiaojuan Chen, Wenyu Yang, Ye Guo, Li Zhang, Yao Zou, Yumei Chen, Yingchi Zhang, Min Ruan, Xiaofan Zhu

**Affiliations:** ^1^ Department of Pediatric Hematology, State Key Laboratory of Experimental Hematology, National Clinical Research Center for Blood Diseases, Haihe Laboratory of Cell Ecosystem, Institute of Hematology & Blood Diseases Hospital, Chinese Academy of Medical Sciences & Peking Union Medical College, Tianjin, China; ^2^ Department of Hematology/Oncology, Guangzhou Women and Children’s Medical Center, Guangzhou Medical University, Guangzhou, China; ^3^ Department of Hematology, The First Affiliated Hospital of Zhengzhou University, Zhengzhou, China

**Keywords:** non-DS-AMKL, pediatric, treatment, outcomes, prognostic factors

## Abstract

Non-Down’s syndrome acute megakaryocytic leukemia (non-DS-AMKL) is a subtype of childhood acute myeloid leukemia (AML), whose prognosis, prognostic factors and treatment recommendations have not yet to be defined in children. We conducted a retrospective study with 65 newly diagnosed non-DS-AMKL children from August 2003 to June 2020 to investigate the clinical impact of factors and clinical outcome. Among all 65 patients, 47 of them were treated at our center who received three different regimens due to time point of admission (CAMS-another, CAMS-2009 and CAMS-2016 protocol), and the efficacy were compared. Patients with newly diagnosed non-DS-AMKL accounted for 7.4% of pediatric AML cases. The median age of the patients was 18 months at diagnosis, and over 90% of them were under three-years-old. The overall survival (OS) rates were 33.3% ± 1.7%, 66.7% ± 24.4% and 74.2% ± 4.0% for three groups (CAMS-another, CAMS-2009 and CAMS-2016 regimen), respectively. In CAMS-2016 group, the complete remission (CR) rate after induction was 67.7% (21/31), while the total CR rate after all phases of chemotherapy was 80.6% (25/31). The 2-year survival probability did not significantly improve in patients underwent HSCT when compared with non-HSCT group (75.0% ± 4.7% vs. 73.9% ± 4.6%, *p*=0.680). Those who had a “dry tap” during BM aspiration at admission had significantly worse OS than those without “dry tap” (33.3% ± 8.6% vs. 84.0% ± 3.6%, *p=*0.006). Moreover, the results also revealed that patients with CD34+ had significantly lower OS (50.0% ± 6.7% vs. 89.5% ± 3.5%, *p=*0.021), whereas patients with CD36+ had significantly higher OS than those who were negative (85.0% ± 4.0% vs. 54.5% ± 6.6%, *p*=0.048). In conclusion, intensive chemotherapy resulted in improved prognosis of non-DS-AMKL children and subclassification may base on “dry tap” and immunophenotypic. Although some progress has been made, outcomes of non-DS-AMKL children remain unsatisfactory, especially in HSCT group, when compared with other AML types.

## Introduction

Acute megakaryoblastic leukemia (AMKL) was first described in 1931 by Von Boros (1). In 1978, Breton-Gorius et al utilized immunoelectron microscopy (IEM) to show that the blast cells of AMKL patients were with positive reactions for platelet peroxidase (PPO) (2). AMKL is a subtype of acute myeloid leukemia (AML) classified as megakaryocyte lineage (M7) by the French-American-British (FAB) cooperative group classification system of hematological neoplasias in 1985 (3). According to recent studies, the proportion of AMKL is around 1% in all adult AML patients (4, 5), while the incidence of AMKL in AML children ranges from 4% to 15% (6–12). Identification of markedly decreased CD41 (GPIIb) and CD61 (GPIIIa) expression levels, which are diagnostic for AMKL patients (13). There are also likely to be many false positives in the result of flow cytometry due to bone marrow (BM) aspiration may be difficult allowing for extensive myelofibrosis caused by megakaryocytes, known as “dry tap”. These increase the difficulty of diagnosis and lead to misdiagnosis. In view of the documented diagnostic bias for the reason above, elimination of misdiagnosis and improve of prognosis may be warranted.

AMKL can be divided into two subgroups in pediatrics: AMKL with Down syndrome (DS-AMKL) and without Down syndrome (non–DS-AMKL). AMKL is the most common kind of AML in children with Down syndrome, and the prognosis of DS-AMKL is better than non–DS-AMKL (14, 15). When comapred with DS-AMKL, non–DS-AMKL may be with biologically heterogeneity, and the prognosis of non–DS-AMKL was thought to be poor (6, 15, 16). However, in view of the low incidence of this type of childhood AML, the prognosis and potential risk factors for Non-DS-AMKL remains debatable (7, 11, 17, 18). The purpose of this study was to determine the prevalence, clinical symptoms at presentation, hematologic, immunophenotypic, cytogenetic, and molecular characteristics of childhood non–DS-AMKL. Furthermore, we analyzed the prognosis and evaluated the potential risk factors of these patients.

## Patients and methods

### Patients

We reviewed data from 65 patients with newly diagnosed non-DS-AMKL at the Institute of Hematology & Blood Diseases Hospital between August 2003 and June 2020. Patients with non-DS-AMKL were ≤16 years old. The diagnosis of AMKL was established based on the 2016 WHO categorization criteria (19). Diagnostic criteria of AMKL met one or more following criteria: 1) The BM aspirate exhibited a blast cell infiltrate that comprised ≥20% of all cells, and with >50% of the blast cells being identified as megakaryoblasts; 2) the expression of CD41, CD42b and/or CD61 was positive, as demonstrated by flow cytometry with monoclonal or polyclonal platelet-specific antibodies; 3) In cases with BM “dry tap” or myelofibrosis, a BM clot or biopsy was necessary, and the cell of origin was required to be identified as part of the megakaryocyte lineage. Positive immunocytochemical staining for platelet-specific antigens such as factor VIII, CD41, CD42b and CD61 revealed this; 4) In the absence of immunophenotyping or biopsy, the diagnosis was confirmed by electron microscopic identification of PPO activity or immunocytochemical staining for platelet-specific antigen CD41 positive in BM or peripheral blood samples, or both in blasts cells (3, 20). Immunophenotyping or immunohistochemistry should always be used to confirm the diagnosis (6). Exclusion criteria included DS-AMKL and AMKL as a secondary malignancy. Cytogenetic studies and Next-generation sequencing (NGS) were performed in some cases. This study was approved by our institution’s ethical committee. Consent was obtained from all patients’ parents or guardians.

### Treatment protocols

During the study, three different treatment protocols were used. Six patients were treated according to the Chinese Academy of Medical Science (CAMS)-another protocol, nine patients received CAMS-2009 protocol (21), and thirty-one patients received CAMS-2016 protocol. The CAMS-2016 of non-DS-AMKL regimen consists of induction and consolidation treatment. If the white blood cell (WBC) count was ≥4×10^9^/L or associated with BM hyperactivity, the standard induction treatment regimen was used, included: etoposide, 150 mg/m^2^ with a 2-hour infusion on days 1-5, idarubicin, 8 mg/m^2^ with a 1-hour infusion on days 6-8 (mitoxantrone 5 mg/m^2^ days 6-10, early availability), and cytarabine, 200 mg/m^2^ with a 12-hour infusion on days 6-12. If the WBC count is less than 4×10^9^/L and the degree of BM hyperplasia is less than active, the standard induction treatment regimen consisted of homoharringtonine 1 mg/m^2^/d, cytarabine 10 mg/m^2^/d, q12h, and granulocyte colony stimulating factor (G-CSF) 200ug/m^2^/d (mix 200ug/d, if WBC ≥20×10^9^/L, stop it) on days 1-14. In patients with severe infections, it can be reduced to 10 days. If CR was not achieved, a second course of induction therapy was administered. High-dose cytarabine combined with etoposide or idarubicin/mitoxantrone was used in the five courses of consolidation treatment. In the consolidation treatment, the course and dosage of medium and large doses of cytarabine have been increased. HSCT is recommended for high-risk patients with relapsed or refractory disease or high minimal residual disease (MRD). If HSCT was not feasible, consolidation and strengthening treatment should be continued. Intrathecal multi-drug chemotherapy was used once per course of treatment to provide prophylactic treatment for the central nervous system.

### Definition and statistical analysis

CR was defined as BM with <5% blasts and evidence of normal hematopoietic cell regeneration. Early death was defined as an event that occurred within 30 days of a diagnosis. The study’s primary endpoints were event-free survival (EFS) and overall survival (OS). EFS was defined as the time from diagnosis to the first event, which included failure to achieve remission, relapse, secondary malignancy, being lost to follow-up, or death from any cause. OS was defined as the time of death from any cause. Categorical variables are expressed as sums and percentages of total numbers. Since continuous variables are not normally distributed, median, minimum, and maximum values were utilized as descriptive statistics. To analyze the differences in continuous variables, a non-parametric test (Mann-Whitney U test) was used, and frequencies were analyzed using Fisher’s exact test. The Kaplan-Meier survival analysis was used to estimate the 2-year probabilities of EFS and OS, and the log-rank test was used to compare survival. Bonferroni-adjusted log-rank tests were conducted to assess differences in separated groups, and the significance level was 0.017 after Bonferroni correction for multiple analysis. A multiple Cox regression model was used to perform multiple regression analysis on EFS and OS. All variables with a *P*<0.10 in univariate analysis were included in the multivariate analysis in logistic regression model. A two-sided *P*-value of <0.05 was deemed to be statistically significant. All clinical statistical analyses were performed using SPSS 25.

### Next-generation sequencing

The DNA from the BM of the patients was extracted using the QIAamp DNA Mini kit (QIAGEN) and purified with the Twist Binding and Purification Beads Kit (Twist Bioscience) following the manufacturer’s instructions. Then, using the Twist Fast Hybridization Target Enrichment protocol, target genes were enriched, amplified, and purified. The Illumina NovaSeq 6000 platform was used to sequence the target-enriched DNA libraries, with an average sequencing depth of 1000×. After quality control of the FASTQ files by FastQC (V 0.11.5), the reads were aligned to the reference genome (hg19) using BWA (V 0.7.10), sorted with SAMtools (V 0.1.19), and deduplicated with Picard (V 1.123). Somatic mutations were then detected with Pisces (V 5.1.6.54) and annotated with ANNOVAR.

## Results

In this study, we included 65 non-DS-AMKL patients between January 2003 and June 2020, accounting for 2.1% (65/3034) of newly diagnosed acute leukemia and 7.4% (65/876) of AML (including AML-M3 patients) in our center. **Table 1** showed the baseline characteristics of all 65 included non-DS-AMKL patients. The median age at diagnosis was 18 months (ranging from 5 to 89 months), and 59 cases (90.8%) were ≤3-years-old.

**Table 1:** Baseline characteristics of included non-DS-AMKL patients (n = 65).

Characteristics	Patients
Gender ratio	43M/22F
Median age at diagnosis, months(range)	18 (5-89)
Median time from onset to diagnosis(range)	2 (0.2-7)
Median WBC count, ×10^9^/L (range)	11.58 (2.44-55.35)
Median Hb count, g/L (range)	82.4 (27-129)
Median PLT count, ×10^9^/L (range)	32 (6-222)
Hepatosplenomegaly, no. (%)	24 (36.9%)
Median BM blasts, % (range)	42.5 (4.0 -97.0)
Median PB blasts, % (range)	16 (0 - 81.0)
“Dry tap”, no. (%)	19/65(29.2%)
**PPO (n=47), no. (%)**	
PPO positive	39 (83.0%)
**Immunophenotype features (n=58), no. (%)**	
CD61	28 (48.3%)
CD41	36 (62.1%)
CD42b	25 (43.1%)
CD34	19 (32.8%)
CD36	33 (56.9%)
**Cytogenetic features (n=60), no. (%)**	
complex karyotypes	21 (35.0%)
+21	18 (30.0%)
+19	19 (31.7%)
+8	20 (33.3%)
-7	2 (3.3%)
-13	3 (5.0%)
-15	4 (6.6%)

M, male; F, female; Hb, hemoglobin; PLT, platelet; BM, bone marrow; PB, peripheral blood; PPO,platelet peroxidase.

**Figure 1:**
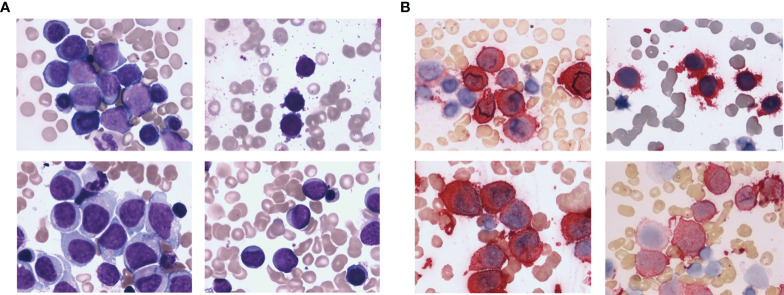
HE staining **(A)** and CD41 immunohistochemical staining **(B)** of the bone marrow from non-DS-AMKL children.

Baseline characteristics was showed in **Table 1**. Anemia, bleeding or fever were initial symptoms in 65 non-DS-AMKL cases in this study. There were 21 cases (32.3%) with pale skin, 38 cases (58.5%) with fever, 31 cases (47.7%) with skin ecchymosis or epistaxis, lymphadenopathy in 2 cases, and bone pain in 4 cases. Physical examination revealed palpable hepatosplenomegaly in 24 cases (36.9%). The morphology of BM varies (**Figure 1A**). The proportion of megakaryocytes stained with CD41 was 39.5% (7%–91%) in BM smears (**Figure 1B**) and 22.5% (2%–59%) in peripheral blood smears. BM biopsy was performed on 12 non-DS-AMKL children, four of whom were CD42b positive. There were six cases of MF-2 and three cases of MF-3. BM clot was performed on 6 non-DS-AMKL children, four of whom were CD42b and/or CD61 positive. Cytogenetic analysis was performed in 60 patients and 21 cases among them was with complex karyotypes.

NGS were performed in 29 patients. 20 of all 29 cases were without disease-related mutations. Three cases carried MPL S505N mutation, as well as one case was with JAK2 V617F and R867Q mutations. The other five cases were with JAK2 M511I, JAK2 V617F, JAK2 R867Q, SUZ12 R286X, and RB1 R255X mutations respectively, and frequency of mutations ranging from 0.85% to 27.8%.

## Outcomes

### Prognosis of patients received three protocols

**Figure 2:**
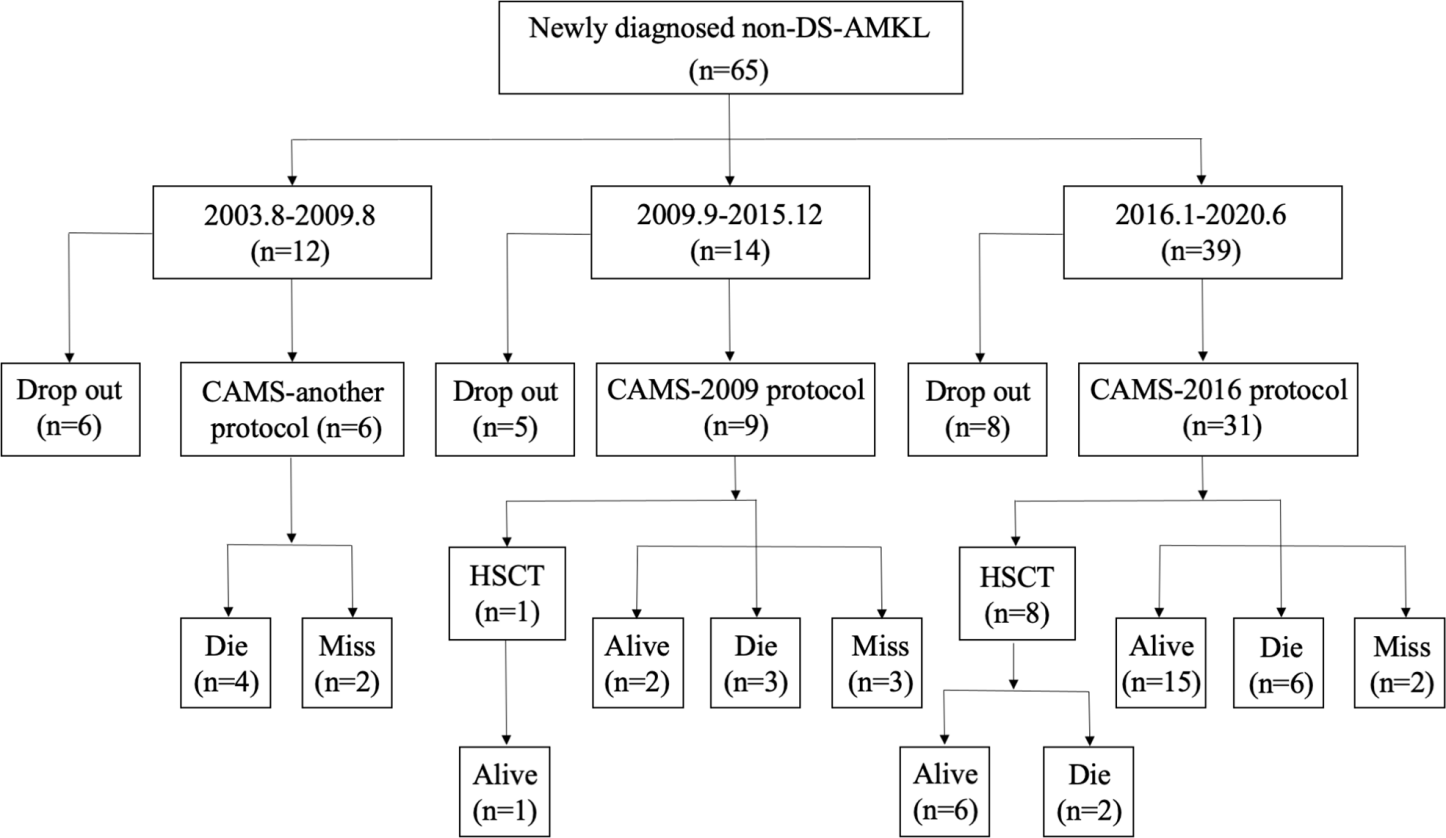
A flowchart depicting newly diagnosed cases of non-DS-AML at our center from August 2003 to June 2020.

The treatment regimens were classified into three groups: previous treatment (from August 2003 to August 2009), the CAMS-2009 regimen (from September 2009 to December 2015), and the CAMS-2016 regimen (from January 2016 to June 2020). Among all 65 patients, 47 of them were treated who received three different regimens due to time point of admission (CAMS-another, CAMS-2009 and CAMS-2016 protocol). The baseline characteristics between patients who underwent treatment and dropout were compared, and there exist no difference between two groups (**Supplementary Table 1**). The percentage of non-DS-AMKL children who dropped out of treatment gradually decreased from 50.0% (6/12) to 20.5% (8/39) (**Figure 2**).

**Figure 3:**
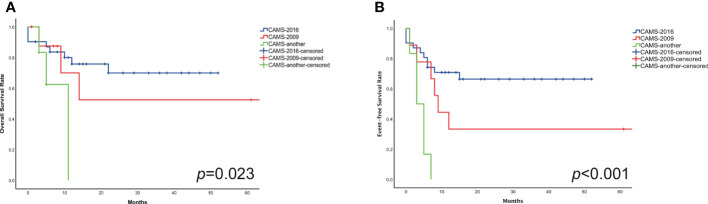
Compared the OS **(A)** and EFS **(B)** in three distinct subgroups of pediatric non-DS-AMKL.

The estimated 2-year probability of OS rates in three different subgroups (CAMS-another, CAMS-2009, and CAMS-2016 regimen) were 33.3% ± 1.7%, 66.7% ± 24.4%, 74.2% ± 4.0%, respectively (*p*=0.023). The difference between CAMS-another and CAMS-2016 protocol was statistically significant (*p*=0.007). However, there was without statistical significance between CAMS-another and CAMS-2009 regimen (*p*=0.101), nor CAMS-2009 and CAMS-2016 regimen (*p*=0.543) (**Figure 3A**).

The estimated 2-year probability of EFS rates in three different subgroups (CAMS-another, CAMS-2009, and CAMS-2016 regimen) were estimated to be 0.0% ± 0.9%, 33.3% ± 19.2%, 67.7% ± 4.1%, respectively (*p*<0.001). The difference between CAMS-another and CAMS-2009 regimen, as well as CAMS-another and CAMS-2016 regimen, was statistically significant (*p*=0.011, *p*<0.001, respectively). There was no significant difference between CAMS-2009 and CAMS-2016 regimen (*p*=0.113) (**Figure 3B**).

### The prognosis and risk factors in CAMS-2016 protocol

For the credibility of the analysis (22, 23), only 31 patients received CAMS-2016 protocol was considered for the further analysis of prognostic factors (**Supplementary Table 2**). The median time of follow-up was 16.1 months (range, 4.6-71.8 months). Three children died as a result of a severe infection, gastrointestinal bleeding, and multiple organ failure during early induction chemotherapy. Three cases remained not remission (NR). In CAMS-2016 group, the complete remission (CR) rate after induction was 67.7% (21/31), while the total CR rate after all phases of chemotherapy was 80.6% (25/31). During the induction, 85.7% of patients with CR survived, and eight patients experienced MRD-negative remission while seven children were still alive in the first CR after induction.

**Figure 4:**
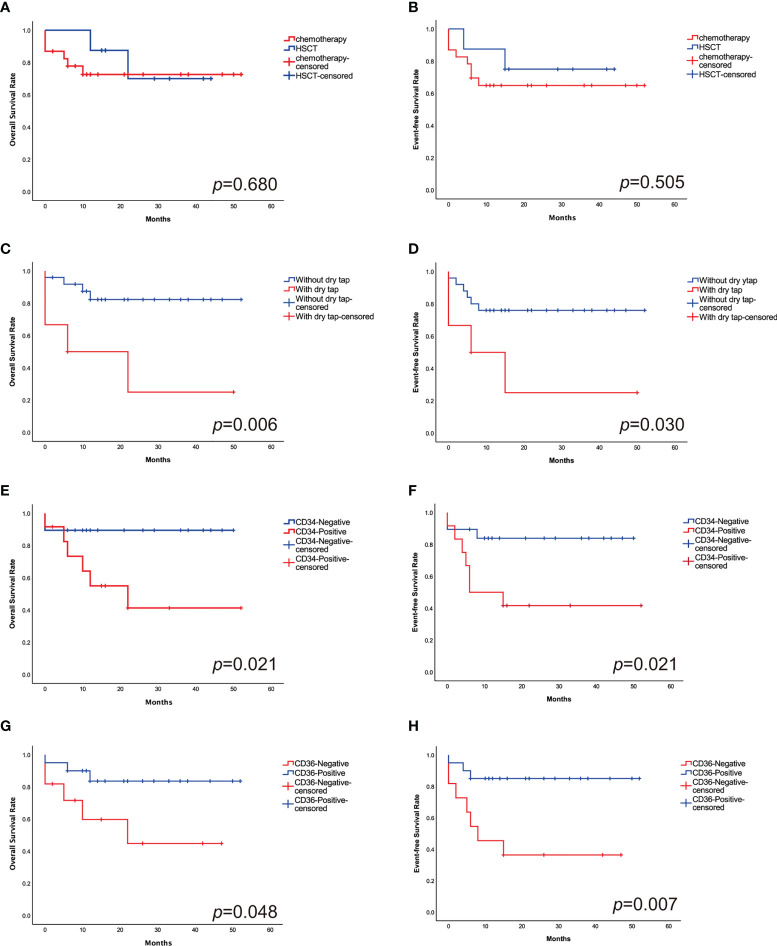
The 2-year probabilities of OS **(A)** and EFS **(B)** comparing the outcomes in HSCT cohort with chemotherapy cohort. There is no significant difference in outcomes between the study groups. The 2-year probabilities of OS **(C)** and EFS **(D)** comparing the outcomes of patients with and without “dry tap”. The 2-year probabilities of OS **(E)** and EFS **(F)** comparing the outcomes of CD34+ and CD34- patients. “Dry tap” and CD34+ confer a poor outcome. The 2-year probabilities of OS **(G)** and EFS **(H)** comparing the outcomes of CD36+ and CD36- patients. CD36+ have a favorable outcome compared with CD36-.

The estimated 2-year probability of OS and EFS was 74.2% ± 4.0% and 67.7% ± 4.1%. 23 patients received intensive chemotherapy and eight patients received HSCT. In chemotherapy cohort, the 2-year OS and EFS was 73.9% ± 4.6% and 65.2% ± 4.9%, respectively, and in transplantation cohort, they were 75.0% ± 4.7% and 75.0% ± 5.4% (**Figures 4A, B**). The OS and EFS rates were similar in both cohorts.

Patients who had “dry tap”, which indicated the possibility of myelofibrosis, had significantly worse OS and EFS than those who without (33.3% ± 8.6% vs. 84.0% ± 3.6%, *p*=0.006; 33.3% ± 8.8% vs. 76.0% ± 4.1%, *p*=0.030, respectively) (**Figures 4C, D**). CD34+ patients have lower 2-year OS and EFS rates than CD34- patients (50.0% ± 6.7% vs. 89.5% ± 3.5%, *p*=0.021; 41.7% ± 6.7% vs. 84.2% ± 4.0%, *p*=0.021, respectively) (**Figures 4E, F**). Patients who are CD36+ have superior 2-year OS and EFS rates than CD36- patients (85.0% ± 4.0% vs. 54.5% ± 6.6%, *p*=0.048; 85.0% ± 3.9% vs. 36.4% ± 6.2%, *p*=0.007, respectively) (**Figures 4G, H**) (**Table 2**).

**Table 2:** Effects of potential factors on clinical outcomes in CAMS-2016 protocol (n=31).

		Cases	OS, %	*P* value	EFS, %	*P* value
**Gender**					
male	22		77.3% ± 4.4%	0.426	72.7% ± 4.7%	0.302
female	9		66.7% ± 7.9%		55.6% ± 8.0%	
**Age**						
≤12month	8		87.5% ± 5.5%	0.401	62.5% ± 7.5%	0.699
>12month	23		69.6% ± 4.7%		69.6% ± 4.7%
**Dry tap**						
Y	6		33.3% ± 8.6%	0.006	33.3% ± 8.8%	0.030
N	25		84.0% ± 3.6%		76.0% ± 4.1%
**Immunophenotype**						
**CD34**						
positive	12		50.0% ± 6.7%	0.021	41.7% ± 6.7%	0.021
negative	19		89.5% ± 3.5%		84.2% ± 4.0%
**CD36**						
positive	20		85.0% ± 4.0%	0.048	85.0% ± 3.9%	0.007
negative	11		54.5% ± 6.6%		36.4% ± 6.2%
**CD41**						
positive	18		77.8% ± 4.6%	0.779	77.8% ± 4.6%	0.238
negative	13		69.2% ± 5.9%		53.8% ± 6.4%	
**CD61**						
positive	15		80.0% ± 2.6%	0.661	80.0% ± 2.6%	0.215
negative	16		68.8% ± 5.4%		56.3% ± 5.9%	
**CD42b**						
positive	16		81.3% ± 4.7%	0.529	81.3% ± 4.7%	0.171
negative	15		66.7% ± 5.6%		53.3% ± 6.0%	
**Cytogenetic**						
**complex karyotypes**						
Y	11		63.6% ± 3.9%	0.222	63.6% ± 3.7%	0.599
N	20		80.0% ± 4.4%		70.0% ± 4.9%
**Trisomy 8 and/or Trisomy 19 and/or Trisomy 21**						
Y	14		57.1% ± 6.7%	0.057	57.1% ± 6.8%	0.233
N	17		88.2% ± 3.9%		76.5% ± 4.8%

N, no; Y, yes.

Multivariate analysis demonstrated a trend toward a poor prognosis for patients with “dry tap” (*p*=0.064) and CD34+ (*p*=0.096). Patients with CD36+ demonstrated a trend toward a favorable prognosis in EFS (*p*=0.054). In the univariate analysis, they were statistically significant (**Table 3**).

**Table 3:** Multivariable Cox Regression Analysis for OS and EFS in CAMS-2016 protocol n = 31.

	OS			EFS		
	HR	95% CI	*P* value	HR	95% CI	*P* value
Dry tap	3.970	(0.925,17.051)	0.064	2.646	(0.723,9.683)	0.142
CD34-positive	4.038	(0.781,20.865)	0.096	3.006	(0.741,12.194)	0.124
CD36-positive	0.422	(0.093,1.919)	0.264	0.256	(0.064,1.026)	0.054

There were insignificant differences in the outcomes of megakaryocytic differentiation-related antibodies (CD41, CD42b, and CD61). The clinical characteristics of the patients, such as gender and age, had no effect on survival as well as complex karyotypes (**Table 2**).

## Discussion

This is a single-center retrospective study to report on the clinical characteristics, outcomes, and potential prognostic factors of newly diagnosed non-DS-AMKL in children. In our study, only 7.4% of pediatric AML cases were diagnosed as non-DS-AMKL. However, the diagnosis of AMKL is frequently challenging due to a high incidence of myelofibrosis, resulting in the failure of BM aspiration. This complicates the diagnosis of AMKL. In our clinical practice, PPO activity on electron microscopy (2, 20), or immunocytochemical staining for platelet-specific antigen CD41 positive in BM or peripheral blood samples, or both in blast cells (24, 25), was recommended for diagnosis when blasts in BM were <20% or absence of immunophenotyping or biopsy in non-DS-AMKL. What’s more, immunophenotyping or immunohistochemistry may be also warranted to aid in diagnosis, which contributed to increased diagnostic capability in recent years (6).

Due to lack of consensus on treatment recommendations for non-DS-AMKL, children with non-DS-AMKL still experienced a poor prognosis and the survival rates vary substantially between studies (10%–70%) (6, 11, 26). This was one of the reasons for the high dropout rate at our center previously. Hence, more intensive induction and consolidation regimens were adopted in our center. By incorporating idarubicin, high-dose cytarabine and mitoxantrone into the protocol, 5-year OS in German AML-BFM (Berlin-Frankfurt-Münster)-04 of pediatric non–DS-AMKL improved to 70% ± 6% (11). Another Japanese study estimated the 10-year OS rate for patients with non-DS-AMKL to be 76% (7). Based on the findings of the preceding investigation, we added mitoxantrone, idarubicin, etoposide and high-dose cytarabine into our CAMS-2009 regimen. On the top of CAMS-2009, CAMS-2016 incorporated homoharringtonine into the protocol which results in a better prognosis of these patients during the past two decades, which was similar to the earlier studies (7, 11). Moreover, several steps have been taken to facilitate the diagnosis and optimize the treatment of childhood AML in recent years, which may also benefit these patients.

According to the discriminating educated degree and economic status of different family, some of the parents were reluctant to let their children to receive chemotherapy because of financial constraints. The dropout rate was higher during the CAMS-2009 or CAMS-another treatment. In recent years, with a number of measures were introduced, increasing number of children suspected of non-DS-AMKL were diagnosed and the clinical outcome of these patients were also improved. With the optimization of the chemotherapy regimen in our center during the past two decades, the prognosis has increased gradually and very few patients abandoned the treatment in recent years.

In view of the poor prognosis of non-DS-AMKL patients, Garderet et al. recommended allogeneic HSCT in the first CR in this cohort (17). However, the benefits of allo-HSCT continue to be inconclusive, for the small number of AMKL patients who received allogeneic HSCT in CR1 (18, 27). Several studies achieved superior survival rates with intensive chemotherapy alone, with no benefit observed when HSCT was used during post-remission treatment (7, 11). In our study, the estimated 2-year OS for patients with non-DS-AMKL was 73.9% ± 4.6% in chemotherapy group and 75.0% ± 4.7% in transplantation group (*p=*0.680), and the OS were comparable in comparison to two previous studies (7, 11). In this study, the benefit of HSCT was still not obvious. Additional research is required to develop new and more effective treatment options for these children.

AMKL is frequently associated with myelofibrosis (28), which frequently results in a “dry tap” in the BM aspiration. The cause of BM fibrosis is unknown. Previous research suggested that fibroblast growth may be correlated with the production of growth factors by malignant megakaryocytes and their dissemination into the BM microenvironment (29–31). However, very few study found that “dry tap” is related to the prognosis of non-DS-AMKL children. In our study, patients with non-DS-AMKL who had “dry tap” had significantly worse prognosis than those who did not. The multivariate analysis indicates that “dry tap” may be associated with a poor prognosis, but the difference is not statistically significant due to limited sample size.

In our study, non-DS-AMKL patients who are CD34+ have inferior 2-year OS and EFS rates (*p*=0.021, *p*=0.021, respectively). According to some studies, CD34-positive cells may be early lineage specific progenitors in AML-M7 (32). It explains that high CD34 expression on AMKL blasts indicates that megakaryocytes are more primitive and may be associated with a poor outcome. CD36 (thrombospondin receptor) is generally used as a marker for late differentiation in CD34- megakaryocytes. In our study, non-DS-AMKL patients with CD36+ had significantly higher 2-year OS and EFS rates than patients without (*p*=0.048, *p*=0.007, respectively), which is consistent with previous literature reports (12, 33). CD34+ may be a poor prognostic factor and CD36+ may be a good prognostic factor in univariate analysis. Due to the small number of cases, there is no statistically significant difference in multifactorial analysis. However, in our study, non-DS-AMKL patients who are CD41, CD42b, or CD61 positive had no effect on prognosis.

Non-DS-AMKL cases are characterized by the presence of recurrent translocations (which are absent in DS-AMKL), such as complex karyotype or copy-number abnormalities. In non-DS-AMKL children, abnormal chromosome numbers, particularly +8, +19, +21, were more prevalent than in children with DS-AMKL (7, 34). Furthermore, +8 and/or +19 can be found in MDS and other diseases (35, 36). Due to a few circulating leukemic cells, a “dry-tap” BM aspiration, and BM fibrosis, some newly diagnosed AMKL patients have both BM and peripheral blood blasts ≤19%. AMKL can be distinguished from MDS based on the age of onset, the course of the disease, and immunophenotyping or immunohistochemistry of peripheral blood or BM megakaryoblastic cells.

Hussein et al. reported that MPL W515L mutation occurs in a considerable proportion of AMKL with myelofibrosis that was unrelated to primary myelofibrosis (37). Malinge et al. also described a new gain-of-function MPL T487A mutation in non-DS-AMKL with features comparable to MPL W515 mutation (38). In this study, we did not find MPL T487A or MPL W515 mutation, but three cases of MPL S505N mutation were detected. The role of the MPL S505N mutation in the pathogenesis of AMKL is still unknown. JAK2 V617F mutation is rare in acute leukemias but occur in 2 of 11(18%) patients with AMKL (39). In our study, two individuals with non-DS-AMKL had JAK2 V617F mutation; one of them also had JAK2 R867Q mutation. JAK2 R867Q mutation promoted the expression of proliferation marker and inhibited the differentiation marker in AML cell-line (40). More research is needed to determine whether the other JAK2 M511I, SUZ12 R286X, and RB1 R255X mutations have functions in non-DS-AMKL.

There still exist limitations in this study. Despite our study has been conducted for nearly 20 years, owing to non-DS-AMKL (AML-M7) was a rare subtype of childhood AML, the number of participants engaged in this study is still limited and the results may not be fully elucidated.

## Conclusion

In conclusion, intensive chemotherapy resulted in improved prognosis of non-DS-AMKL children and subclassification may base on “dry tap” and immunophenotypic. Although some progress has been made, outcomes of non-DS-AMKL children remain unsatisfactory, especially in HSCT group, when compared with other AML types.

## Data availability statement

The original contributions presented in the study are included in the article/**Supplementary Material**. Further inquiries can be directed to the corresponding authors.

## Ethics statement

The studies involving human participants were reviewed and approved by The Ethics committee of the Institute of Hematology and Blood Diseases Hospital, Chinese Academy of Medical Sciences and Peking Union Medical College. Written informed consent to participate in this study was provided by the participants’ legal guardian/next of kin.

## Author contributions

AZ and LL conceived and designed the study. AZ and SZ drafted the initial manuscript and analyzed the data. MR, XYC, and CL reviewed the initial manuscript. XZ, MR, and YiZ supervised the work. LC, XJC, WY, YG, LZ, YaZ, and YC collected and provided patient clinical data. XZ and MR assigned the protocol, and critically revised the manuscript for relevant intellectual content. All authors contributed to the article and approved the submitted version.

## Funding

This work was supported by the National Key Research and Development Program of China (2021YFE0106900) and the CAMS Innovation Fund for Medical Sciences (CIFMS) (2020-I2M-C&T-B-087).

## Acknowledgments

All authors express thanks to Dr. Yang Lan, Meihui Yi, Luyang Zhang, Yuli Cai and Jing Feng. We want to acknowledge patients and their families for participating in the follow-up.

## Conflict of interest

The authors declare that the research was conducted in the absence of any commercial or financial relationships that could be construed as a potential conflict of interest.

## Publisher’s note

All claims expressed in this article are solely those of the authors and do not necessarily represent those of their affiliated organizations, or those of the publisher, the editors and the reviewers. Any product that may be evaluated in this article, or claim that may be made by its manufacturer, is not guaranteed or endorsed by the publisher.
