# Supplementary material for: Pediatric non–Down’s syndrome acute megakaryoblastic leukemia patients in China: A single center's real-world analysis
Source: Front Oncol. 2022 Oct 4;12:940725. doi: 10.3389/fonc.2022.940725 (PMC9577933; doi:10.3389/fonc.2022.940725)
Supplement: Supplementary file 1 [file Table_1.docx]

**Supplementary Table 1** **Compared characteristic of pediatric non-DS-AMKL patients (n=65)**

| Characteristics | Received treatment（n=46） | Dropped out  (n=19) | *P* value |
| --- | --- | --- | --- |
| Gender ratio | 32 M /14 F | 11 M /8 F | 0.366 |
| Median age at diagnosis, months (range) | 17(5-89) | 20(10-34) | 0.502 |
| Median time from onset to diagnosis (range) | 1.25(0.2-6.5) | 1(0.2-7) | 0.533 |
| Median WBC count, ×10^9^/L (range) | 10.61(2.5-55.35) | 13.19(2.44-50.4) | 0.395 |
| Median Hb count, g/L (range) | 83.5(27-129) | 81(55-108) | 0.383 |
| Median PLT count, ×10^9^/L (range) | 33(7-222) | 30(6-159) | 0.629 |
| Median BM blasts, % (range) | 44.25(4.0-97.0) | 32(15.0-91.0) | 0.718 |
| Median PB blasts, % (range) | 16 (0-78.0) | 15 (0-81.0) | 0.857 |
| Hepatosplenomegaly | 18 | 6 | 0.566 |
| “Dry tap” | 11 | 8 | 0.142 |

M,male; F,female; Hb, hemoglobin; PLT, platelet; BM, bone marrow; PB, peripheral blood.
