# Supplementary material for: Pediatric non–Down’s syndrome acute megakaryoblastic leukemia patients in China: A single center's real-world analysis
Source: Front Oncol. 2022 Oct 4;12:940725. doi: 10.3389/fonc.2022.940725 (PMC9577933; doi:10.3389/fonc.2022.940725)
Supplement: Supplementary file 2 [file Table_2.docx]

**Supplementary Table 2 Characteristics of pediatric non-DS-AMKL patients in CAMS-2016 protocol (n=31)**

| Patient | Age  (month) | Gender | Peripheral blood | | | | Bone marrow | | | | | | |
| --- | --- | --- | --- | --- | --- | --- | --- | --- | --- | --- | --- | --- | --- |
|  |  |  | WBC count  (×10^9^/L) | Hb  (g/L) | PLT count  (×10^9^/L) | Peripheral blast (%) | Marrow  Blast (%) | Dry tap | | Cycles of  induction | Karyotype | Immune subtypes | |
| 1 | 14 | male | 7.41 | 86 | 18 | 20 | 11 | | Y | 1 | 46,XY,del(11)(p13),add(12)(p13),  del(12)(q24.2),-13,+mar[17]/46,XY[3] | CD33+,CD13+,CD36+,  CD41+,CD61+,CD42b+,  HLA-DR+,CD71+ | |
| 2 | 17 | male | 6.73 | 72 | 50 | 20 | 40 | | N | 1 | 56,XY,+del(1)(p13),+2,+6,+7,+7,+8,  +10,+19,+20,+22[17]/56,XY,+1,  der(1;14)(q10;q10),+2,+6,+7,+7,+8,  +10,+19,+19,+20,+22[1]/46,XY[3] | CD38+, CD33+, CD41+, CD61+,CD42b+,CD36+,  CD71+, HLA-DR+, CD4+ | |
| 3 | 14 | male | 4.81 | 108 | 9 | 3 | 29 | | N | 1 | 51,XY,+6,-9,+21,  +4mar,inc[7]/46,XY[13] | CD33+,CD7+,CD41+,CD61+,  CD42b+,CD38+,HLA-DR+  ,CD36+,CD71+,CD4+ | |
| 4 | 16 | male | 14.86 | 98 | 45 | 11 | 17 | | N | 2 | 44,XY,?t(1;1)(p36.1,p21),-5,-7,-17,  +?der(20)t(1;20)(q21;p12),-22,+mar[15]/46,XY[8] | CD33+, CD41+, CD61+, CD42b+,CD71+,CD4+,  CD7+,CD36+ | |
| 5 | 14 | male | 13.81 | 96 | 102 | 11 | 33.5 | | N | 1 | 56,XY,+2,+2,+6,+7,+8,+13,  +14,+19,+19,+20[5]/46,XY[15] | CD33+,CD36+,CD41+,  CD61+,CD42b+, HLA-DR+,CD38+,CD7+,CD71+,CD4+ | |
| 6 | 7 | male | 19.86 | 120 | 36 | 4 | 79 | | N | 1 | 49,XY,t(2;7)(q31;p22),  +19,+21,+22[12]/50,idem,+10[8] | CD117+, CD38+, CD36+,  CD41+, CD61+, CD42b+,  HLA-DR+, CD33+, CD71+ | |
| 7 | 26 | female | 12.37 | 102 | 17 | 30 | 79 | | N | 1 | 46,XX[20] | HLA-DR+,CD33+,CD42b+, CD34+,CD13+,CD7+,  CD71+,CD123+,CD41+,CD61+ | |
| 8 | 30 | male | 24.8 | 65 | 117 | 42 | 27 | | N | 1 | 46,XY[20] | CD36+,CD38+,CD13+,  CD7+,CD33+,CD34+,  CD117+,HLA-DR+,CD41+,  CD61+,CD42b+,CD123+,  CD11b+,CD56+,CD4+ | |
| 9 | 28 | male | 13.14 | 129 | 197 | 3 | 94.6 | | N | 1 | 46,XY[5] | CD56+,CD34+,CD117+,  CD123+,CD33+ | |
| 10 | 21 | female | 29.66 | 68 | 27 | 9 | 79.5 | | Y | 1 | 34-54,XX,+der(X)t(X;1)(q24;q25),  der(1)?t(1;7)(q21;p13),  der(2)?t(1;2)(q21;p11.2),  +6,+add(7)(p11.2),+8,+10,+19,  add(20)(q13.3),+22,  +mar,inc[cp5]/46,XX[4] | CD34+,CD38+,CD41+,CD61+,  HLA-DR+,CD4+,CD36+ | |
| 11 | 17 | female | 5.72 | 79 | 11 | 11 | 79 | | N | 1 | 51～53,XX,+X,add(1)(q10),  -6,del(7)(p21),  +8,+10,add(11)(q25),  +?add(16)(p13.2),  +19,+21,+22,  +der(?)t(?;6)(?;p11.2)×2,  [cp17]/46,XX[3] | CD36+, CD38+, CD42b+, CD41+, CD61+,CD33+,HLA-DR+ | |
| 12 | 14 | female | 9.8 | 84 | 7 | 5 | 43.5 | | Y | Not evaluated | 46,XX,add(1)(q32),?del(2)(p13),  del(12)(p11.2),add(14)(p11.2),  ?add(22)(q13),inc[2] | CD56+,CD117+, CD13+, CD41+, CD42b+,CD33+ | |
| 13 | 30 | male | 19.71 | 74 | 83 | 16 | 61 | | N | 1 | 46,XY[11] | CD36+,CD117+, CD38+,  CD13+, CD33+, CD7+,  CD42b+, CD41+, CD61+,  CD34+, CD56+, CD11b+, CD4+ | |
| 14 | 9 | male | 10.81 | 94 | 37 | 3 | 21 | | N | 1 | ND | No abnormal phenotype | |
| 15 | 25 | female | 8.61 | 63 | 10 | 17 | 61 | | N | 1 | 42,XX,t(1;17)(q21;p13),  del(8)(q22),-16,-16[1]/  42,XX,-1,del(8).add(12),  der(17)t(1;17),-18,-19,-21[1]/  49,XX,+4,+6,del(8),add(12),+mar[1]/  38,X,-X,-5,del(8),-10,-11,  add(12),-14,-16,-17,-19,-21,  +mar[1]/45,XX,-10,  add(17)(p11),-19,+mar[1]/46,XX[15] | CD33+,CD36+,CD9+,  CD117+,HLADR+,  CD11b+,CD41+,CD61+,  CD38+,CD13+,CD4+ | |
| 16 | 11 | female | 44.41 | 114 | 21 | 62 | 55 | | N | 1 | 46,XX,del(1)(p?34),  ?t(1;7)(q21;p15),  der(4),del(5)(q?34),inc[9]/46,xx[1] | CD33+,CD123+,CD42b+,  CD41+,CD61+,CD36+,  HLA-DR+,CD4+ | |
| 17 | 30 | male | 11.48 | 107 | 35 | 6 | 48.5 | | N | 2 | 47,XY,del(4)(q31),der(13),+21[20] | CD34+,CD117+,HLA-DR+,  CD13+,CD33+,  CD38+,MPO+,CD36+ | |
| 18 | 37 | male | 36.4 | 96 | 10 | 47 | 87 | | Y | 1 | 47,XY,add(1)(q32),+21[11]/46,XY[9] | CD34+,CD117+,CD56+,  CD13+,CD33+,CD123+,  CD19+,CD9+ | |
| 19 | 11 | male | 9.18 | 68 | 14 | 0 | 23 | | N | 1 | 56,XY,+Y,+6,+8,+10,+13,+19,  +21,+21,+2,+22[9]/46,XY[1] | CD33+,CD34+,CD117+,  HLA-DR+, CD38+, CD13+,  CD123+, CD7+, CD36+,  CD42b+, CD41+,CD56+,  CD56+ | |
| 20 | 11 | female | 8.9 | 69 | 20 | 33 | 80 | | N | Not evaluated | 49,XX,add(7)(p?21),+8,+10,+19[20] | CD36+, CD33+,CD13+,  HLA-DR+, CD42b+, CD41+, CD61+,CD34+ | |
| 21 | 22 | male | 12.65 | 107 | 69 | 16 | 21.5 | | N | 1 | 46,XY[20] | CD117+, HLA-DR+, CD33+,  CD7+, CD36+,CD38+,  CD123+, CD4+, CD9+ | |
| 22 | 5 | female | 55.35 | 78 | 55 | 78 | 71 | | N | 1 | 46,XX[20] | CD117+, CD33+, CD123+,  CD34+, CD13+, CD56+,  CD19+,cCD3+,CD38+,  MPO+ | |
| 23 | 18 | male | 2.5 | 109 | 82 | 3 | 56 | | N | 1 | 47,XY,del(5)(p11),+21[6]/47,XY,+21[14] | | CD33+,CD117+, CD38+,  CD11b+, CD123+, CD56+,  CD7+, CD36+,CD13+,  HLA-DR+, CD4+ |
| 24 | 89 | male | 8 | 81 | 7 | 2 | 54 | | N | 1 | 46,XY[17] | No abnormal phenotype | |
| 25 | 13 | male | 7.19 | 93 | 34 | 29 | 42.5 | | N | NR | 46,XY,t(13;17)(q22;q21)[18]/46,XY[2] | HLA-DR+,CD36+,CD33+,  CD38+,CD4+,CD9+ | |
| 26 | 14 | male | 16.31 | 27 | 36 | 20 | 25.5 | | N | NR | 52,XY,-5,+6,+?,+10,+14,+20,+21,  +2mar,inc[6]/46,XY,[14] | CD41+,CD61+,CD42b+,  CD33+,CD81+,CD117+,  CD34+,CD38+ | |
| 27 | 10 | male | 5.67 | 89 | 25 | 2 | 20 | | N | NR | 46,XY,t(1;22)(p13;q13)[4]/46,XY[11] | CD45+,CD117+,CD4+ | |
| 28 | 9 | male | 13.6 | 83 | 32 | 3 | 4 | | N | 3 | 46,XY[6] | No abnormal phenotype | |
| 29 | 13 | female | 10.32 | 97 | 62 | 2 | 29 | | Y | 2 | 46,XX[20] | CD123+,CD38+,CD41+,  CD42b+,HLA-DR+,  CD36+,CD33+,CD4+ | |
| 30 | 17 | male | 10.4 | 62 | 14 | 70 | 97 | | N | 1 | 48-53,XY,+2,+6,+7,  +8,+10,+19×2[cp19]/46,XY[1] | CD9+,CD34+,CD38+,  CD33+,CD56+,CD36+ | |
| 31 | 17 | male | 19.09 | 63 | 42 | 15 | 57 | | Y | Not evaluated | 51-55,XY,+3,+6,+7,  +8,+14,+19,+21,+21,  +22[cp9]/47,XY,+?19[4]/46,XY[7] | No abnormal phenotype | |

**Supplementary Table 2 Characteristics of pediatric non-DS-AMKL patients in CAMS-2016 protocol (n=31)（continued）**

| Patient | Response to induction  therapy | HSCT | Method of transplantation | Current status | Survival  （month） | Cause of induction  failure |
| --- | --- | --- | --- | --- | --- | --- |
| 1 | Y | N |  | Alive | 6 |  |
| 2 | Y | N |  | Alive | 10 |  |
| 3 | Y | N |  | Alive | 11 |  |
| 4 | Y | N |  | Alive | 11 |  |
| 5 | Y | N |  | Alive | 12 |  |
| 6 | Y | N |  | Alive | 14 |  |
| 7 | Y | Y | haplo-HSCT | Alive | 15 |  |
| 8 | Y | Y | haplo-HSCT | Alive | 16 |  |
| 9 | Y | N |  | deceased | 10 |  |
| 10 | Y | N |  | deceased | 6 |  |
| 11 | Y | N |  | Alive | 21 |  |
| 12 | Not evaluated | N |  | deceased | Early death | Fever, Intestinal infection，Hemolysis, liver damage  Hemolysis, liver damage |
| 13 | Y | N |  | Alive | 22 |  |
| 14 | Y | N |  | Alive | 26 |  |
| 15 | Y | N |  | Alive | 26 |  |
| 16 | Y | Y | HLA-matched related HSCT | Alive | 29 |  |
| 17 | Y | Y | haplo-HSCT | deceased | 12 |  |
| 18 | Y | Y | haplo-HSCT | deceased | 22 |  |
| 19 | Y | Y | UCBT | Alive | 33 |  |
| 20 | Not evaluated | N |  | deceased | Early death | Gastrointestinal bleeding, lung infections, intestinal infections, cardiac insufficiency |
| 21 | Y | N |  | Alive | 36 |  |
| 22 | Y | N |  | Alive | 2 |  |
| 23 | Y | N |  | Alive | 38 |  |
| 24 | Y | Y | haplo-HSCT | Alive | 42 |  |
| 25 | NR | Y | haplo-HSCT | Alive | 44 |  |
| 26 | NR | N |  | deceased | 5 |  |
| 27 | NR | N |  | Alive | 8 |  |
| 28 | Y | N |  | Alive | 47 |  |
| 29 | Y | N |  | Alive | 50 |  |
| 30 | Y | N |  | Alive | 52 |  |
| 31 | Not evaluated | N |  | deceased | Early death | Gastrointestinal bleeding, intestinal obstruction, multi-organ failure death |

N: no; Y: yes
